# S11-3 Hurdles and springboards in involving different health care workers with physical activity on prescription: an EUPAP experience

**DOI:** 10.1093/eurpub/ckad133.055

**Published:** 2023-09-11

**Authors:** Luc Lipkens, Karlien Devloo

**Affiliations:** Flanders Institute of Healthy Living NGO; Flanders Institute of Healthy Living NGO

## Abstract

**Purpose:**

In Flanders General Practitioners (GP) can use Physical Activity on Prescription (PAP) since 2017. GPs can start a prescription and refer their patients to a PAP-coach. This PAP-coach finishes off the prescription. The PAP-coach follows the patient and reports back to the GP. The Flemish government pays the largest part of the PAP-coaching. In 2019 a collaboration, funded by EU, between different European regions was set up in order to implement a European Physical Activity on Prescription model (EUPAP). In other countries, other Health Care Workers (HCW) used PAP as well. In 12 pilot regions in Flanders, they were allowed to do so. The purpose was to increase the use of PAP and thus increase the physical activity level of patients.

**Project description:**

Development: starting from the experience with GPs and other European regions, a proposal of implementation was made. This proposal was adapted to the feedback of professional associations of HCW.

**Implementation:**

In the 12 pilot regions, network groups were responsible for the local implementation. They received a proposed plan, information, (promotion) materials and content for the training of HCW in PAP. The professional associations were asked to inform their members about PAP.

**Evaluation:**

After 4 months of implementation the local implementators joined two focus groups about the implementation process and their first impressions. After 12 months of implementation two online questionnaires were distributed: one for patients that uses PAP and one for prescribers that could have been using PAP. The first results indicate that (1) it is difficult to inform HCW, (2) most HCW are positive, (3) HCW start making use of PAP but not as much as hoped by PAP-coaches.

**Scaling up:**

An evaluation report with lessons learnt from the 12 pilot regions will be handed over to the Flemish government for scaling up the use of PAP by HCW.

**Conclusion:**

Reaching more patients to become more active through other HCW, than GPs, was implemented in 12 regions. Hurdles and springboards are identified in order to learn from for scaling this up.

